# Supplementary material for: High-resolution cryo-EM structure of photosystem II reveals damage from high-dose electron beams
Source: Commun Biol. 2021 Mar 22;4:382. doi: 10.1038/s42003-021-01919-3 (PMC7985191; doi:10.1038/s42003-021-01919-3)
Supplement: Supplementary file 2 — Supplementary Information [file 42003_2021_1919_MOESM2_ESM.pdf]

Supplementary Information for

High-resolution cryo-EM structure of photosystem II reveals damage from high-dose electron beams

Koji Kato, Naoyuki Miyazaki, Tasuku Hamaguchi, Yoshiki Nakajima, Fusamichi Akita, Koji Yonekura and Jian-Ren Shen

This file contains Supplementary Figures 1-9 and Supplementary Tables 1-2.

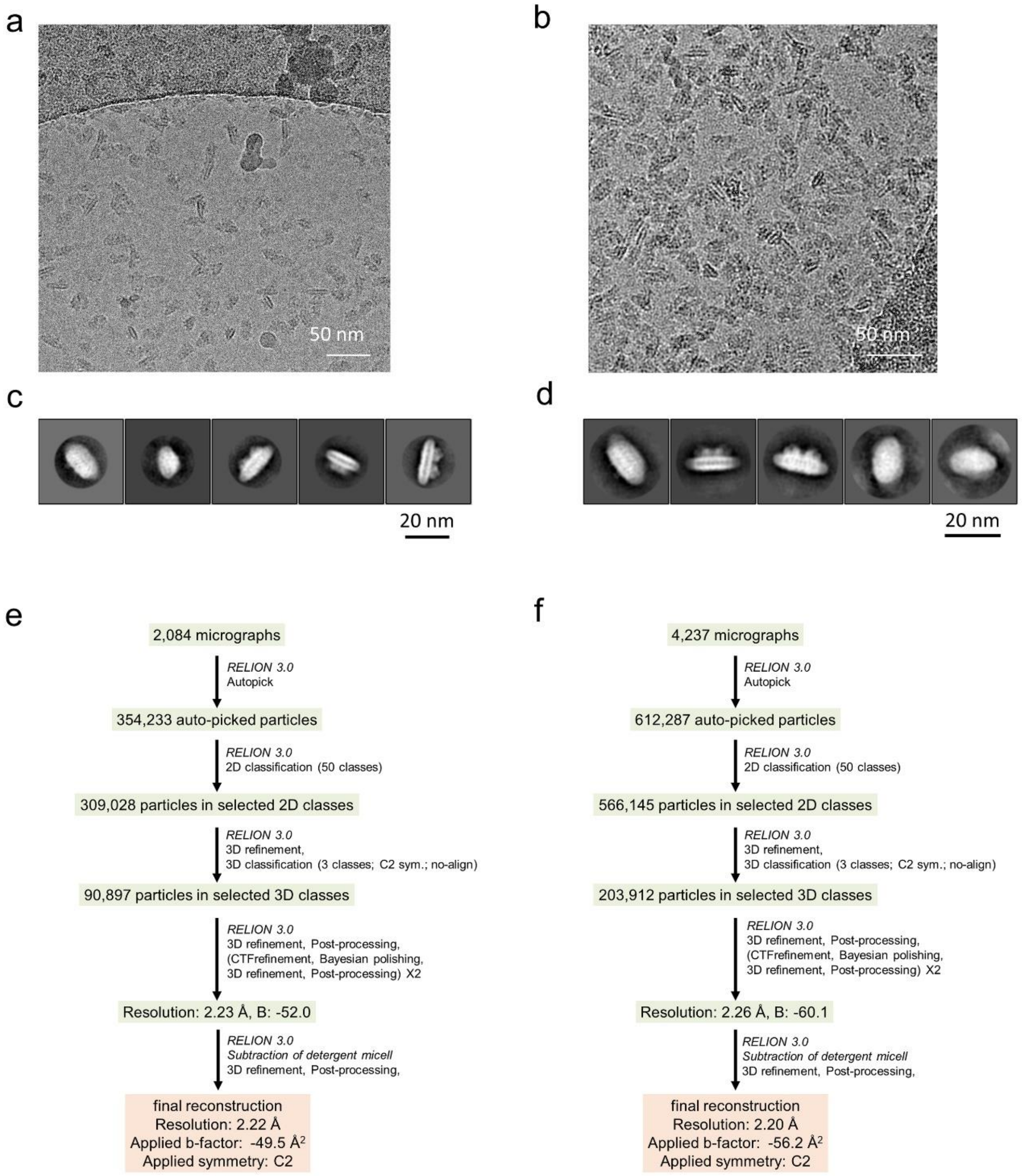

Supplementary Fig. 1 Cryo-EM data collection and processing of PSII from Titan-75k and Titan-96k. **a-b** Representative cryo-EM micrographs of the PSII by Titan-75k (**a**) and Titan-96k (**b**). **c-d** Representative 2D classes of the PSII particles from micrographs taken by Titan-75k (**c**) and Titan-96k (**d**). **e-f** A schematic flowchart showing the classification scheme for PSII from Titan-75k (**e**) and Titan-96k (**f**).

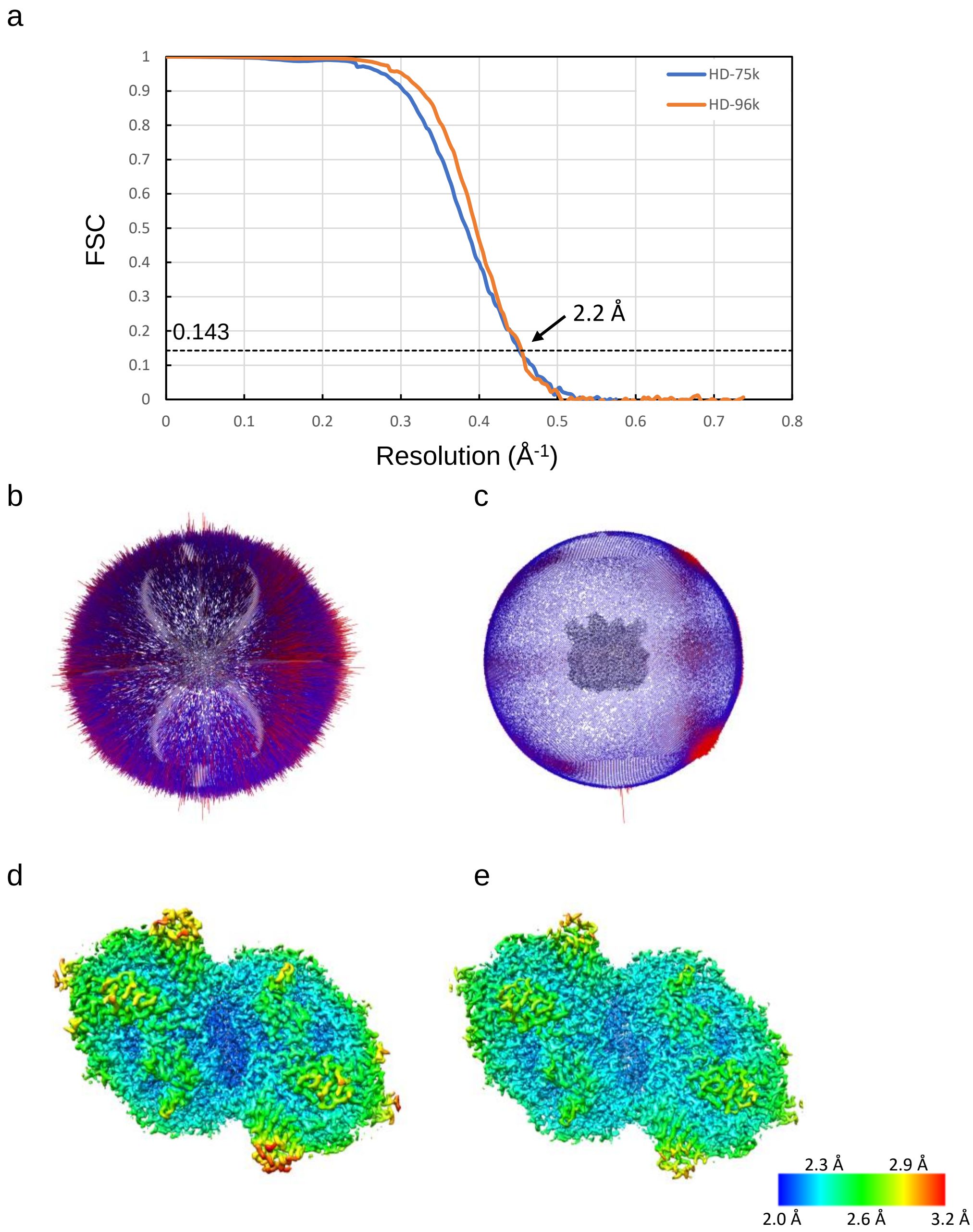

Supplementary Fig. 2 Evaluation of the resolutions of the cryo-EM maps of PSII for the Titan-75k and Titan-96k data sets. a FSC curves of the PSII for high-dose Titan-75k (blue) and high-dose Titan-96k (gray) data sets, calculated between independently refined half maps used for the structure reconstructions. **b-c** Angular distribution of the particles used for reconstruction of the PSII from Titan-75k (**b**) and Titan-96k (**c**). Each cylinder represents one view and the height of the cylinder is proportional to the number of particles for that view. **d-e** Local resolution maps of the PSII from Titan-75k (**d**) and Titan-96k (**e**).

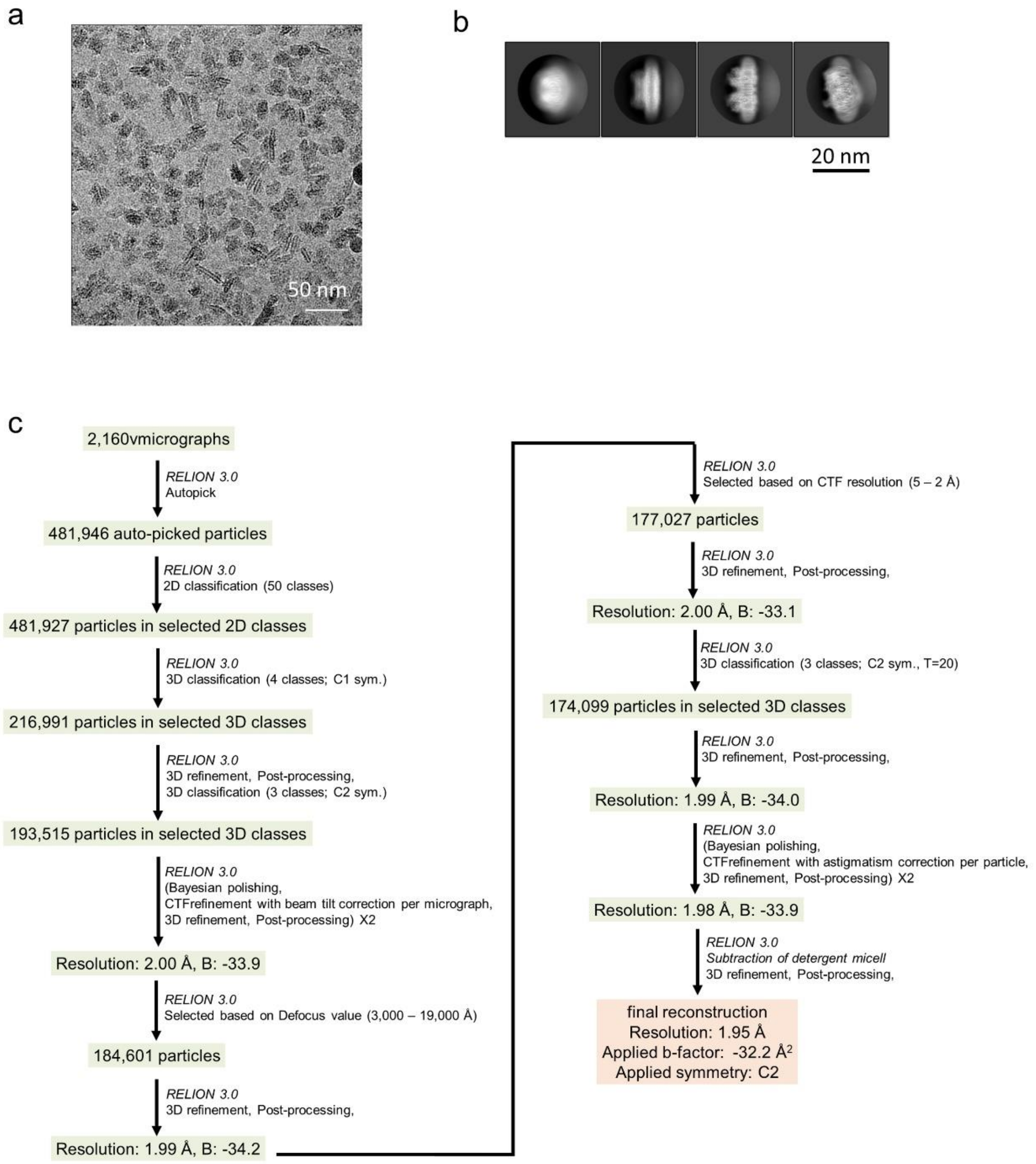

Supplementary Fig. 3 Cryo-EM data collection and processing of PSII by ARM-60k. **a** A representative cryo-EM micrograph of the PSII. **b** Representative 2D classes of the PSII particles. **c** A schematic flowchart showing the classification scheme for the PSII.

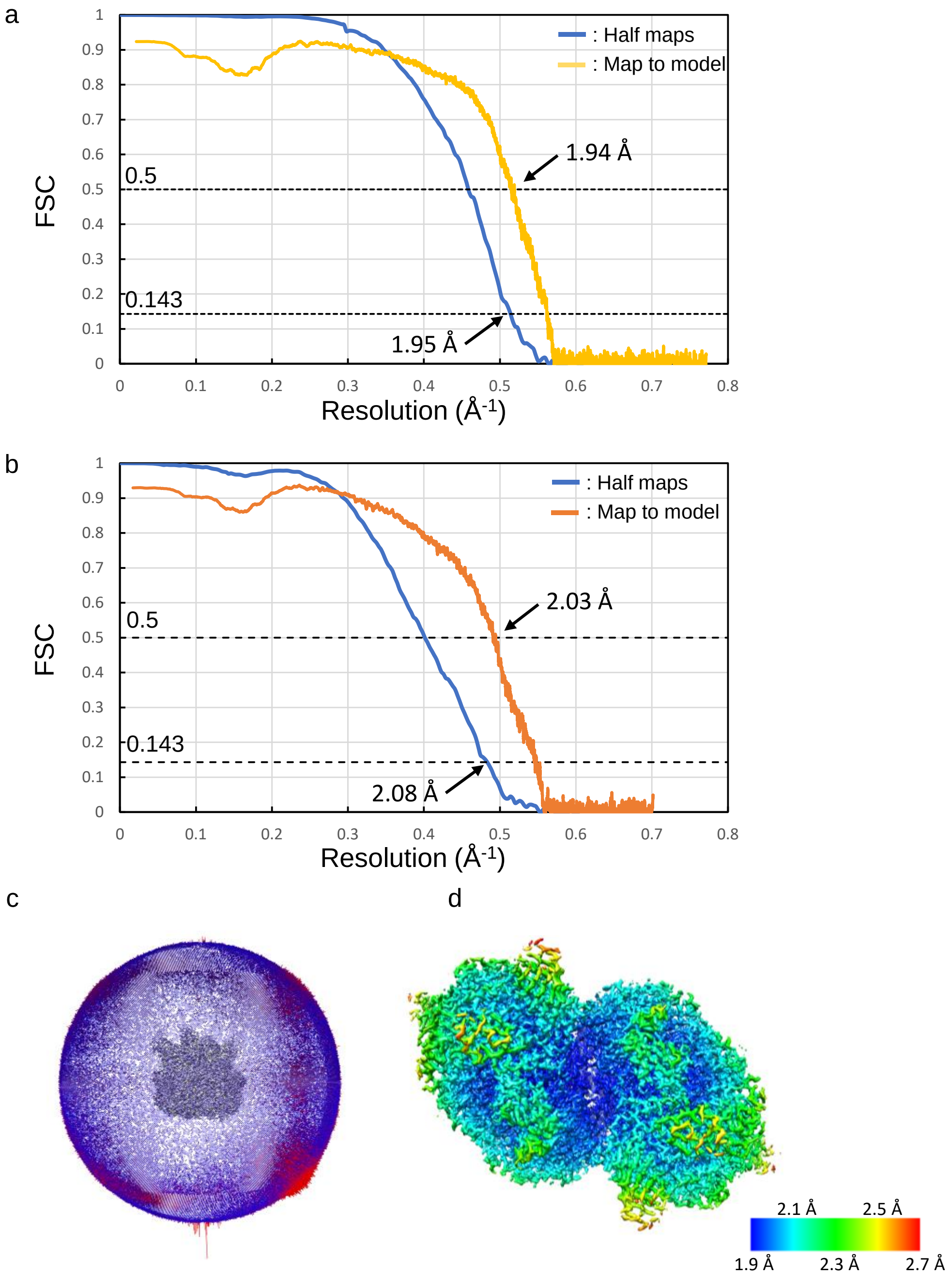

Supplementary Fig. 4 Evaluation of the resolution of the cryo-EM map of PSII for the ARM-60k dataset. a FSC curves of the high-dose PSII for independently refined half-maps (half-maps) and full map vs. model (map to model). **b** FSC curves of the low-dose PSII for independently refined half-maps (half-maps) and full map vs. model (map to model). **c** Angular distribution of the particles used for reconstruction of the high-dose PSII. Each cylinder represents one view and the height of the cylinder is proportional to the number of particles for that view. **d** Local resolution map of the high-dose PSII.

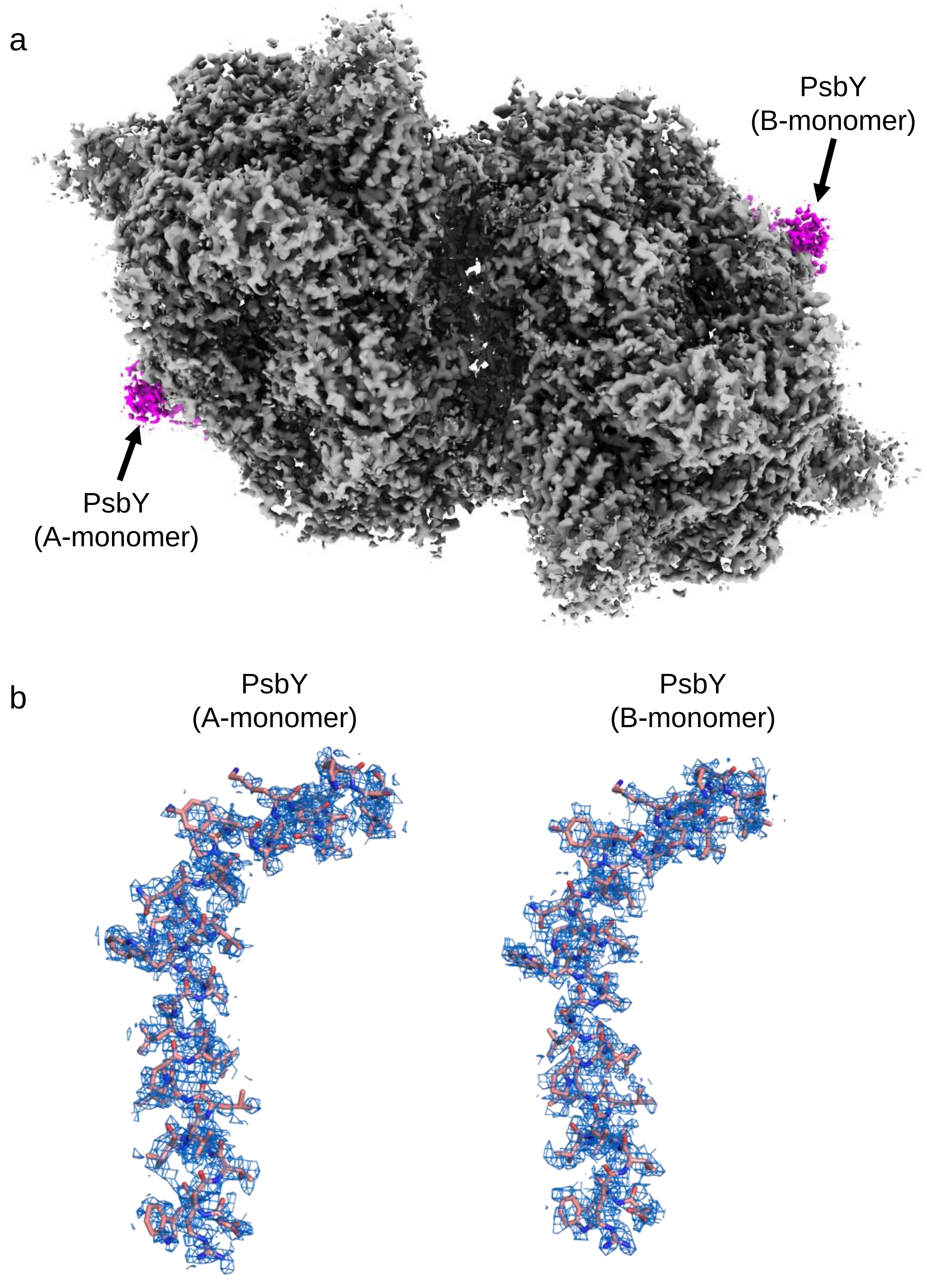

Supplementary Fig. 5 Cryo-EM map of PsbY without C2 symmetry. a The cryo-EM density of the PSII at 2.0 Å resolution, reconstructed with a C1 symmetry. **b** The density of PsbY reconstructed with the C1 symmetry and superimposed with the stick model of PsbY.

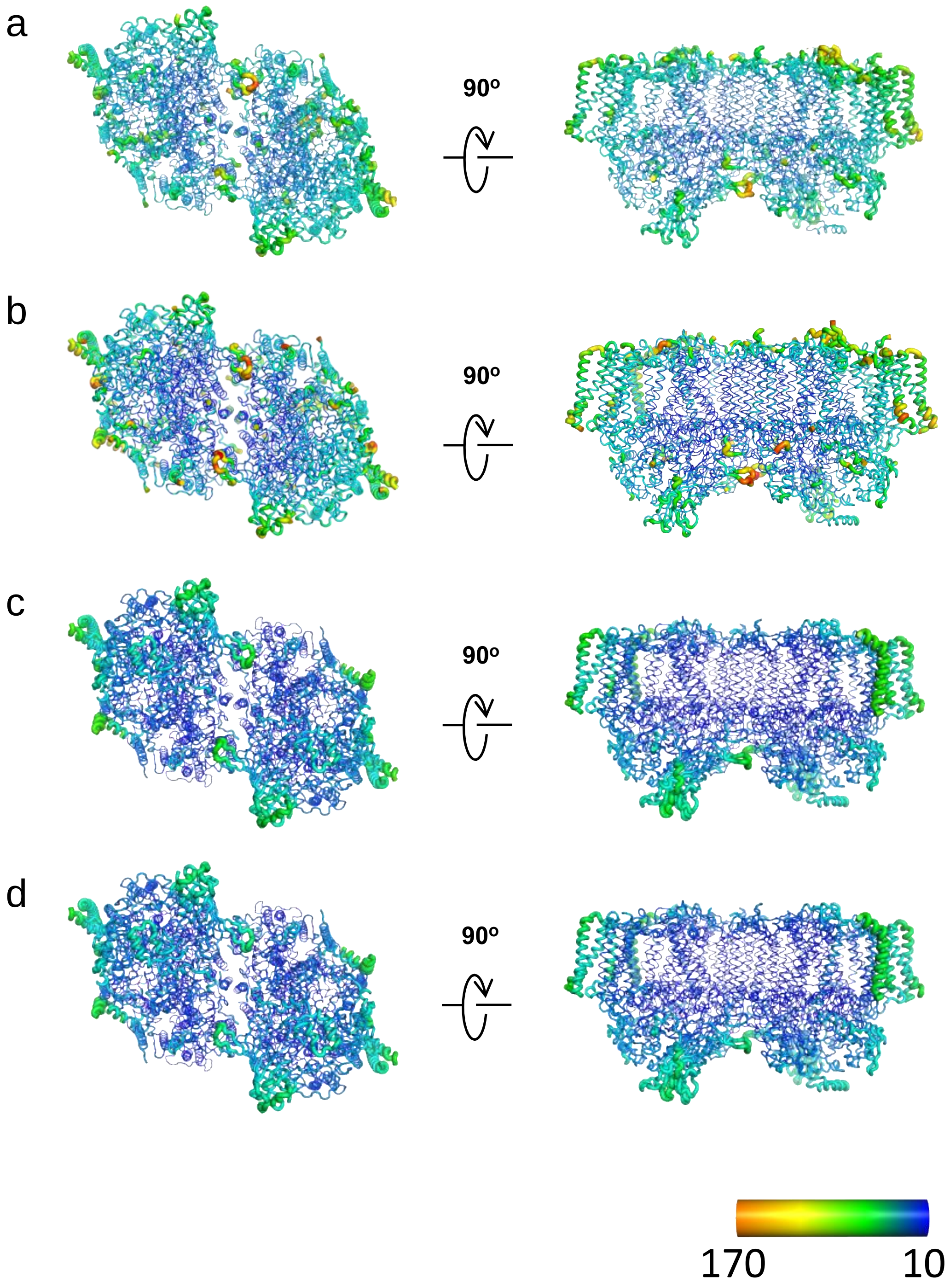

Supplementary Fig. 6 Atomic displacement parameter (ADP) for the crystal structures and cryo-EM structures. a-c Refined ADPs for the SR structure (3WU2) (**a**), XFEL structure (4UB6) (**b**), high-dose of ARM-60k (**c**) and low-dose of ARM-60k data sets (**d**) were shown as heat maps.

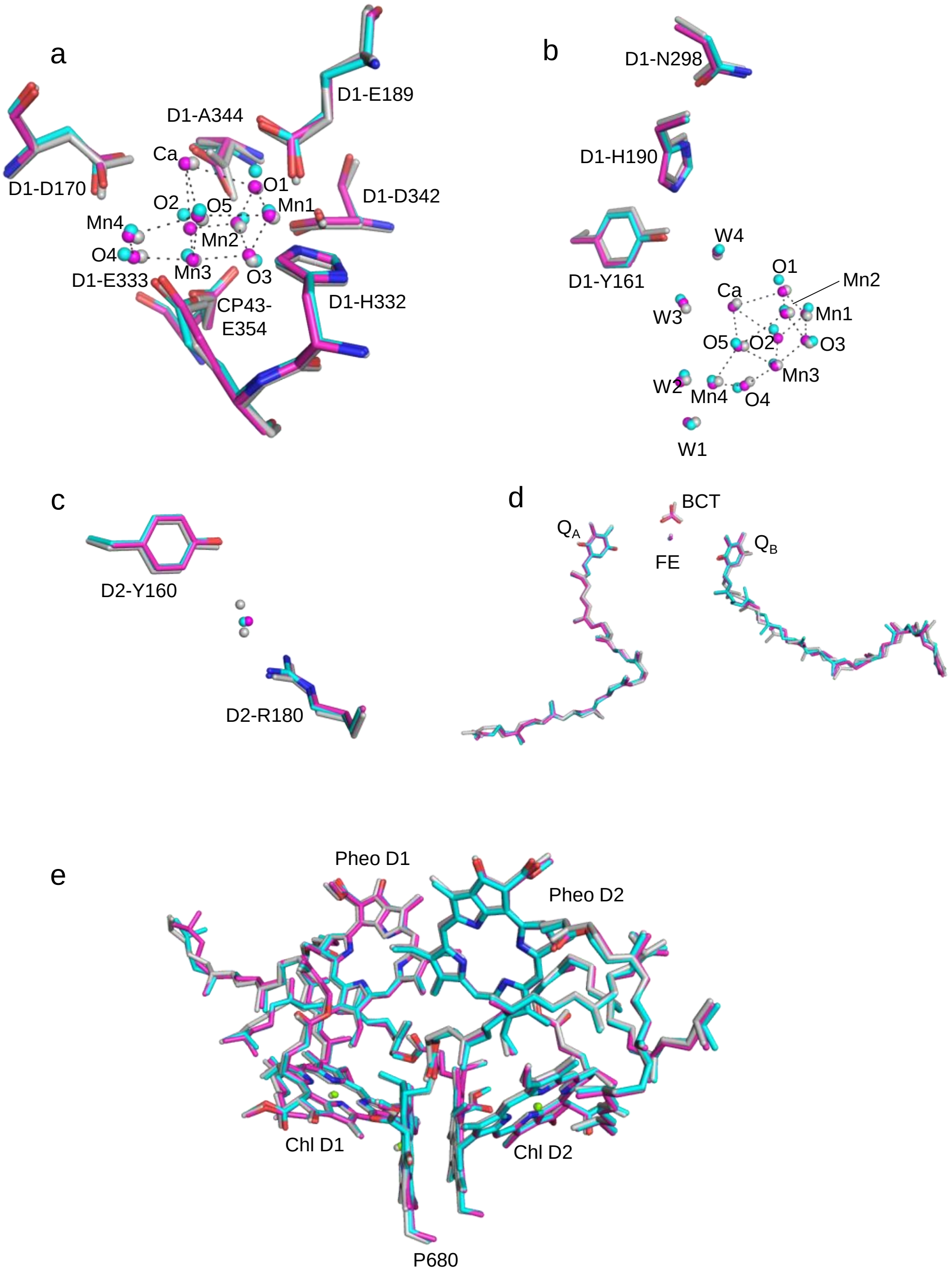

Supplementary Fig. 7 Comparison of high-dose structure (cyan), low-dose structure (magenta) solved by cryo-EM, and the XFEL structure (4UB6) (gray). a The Mn_4CaO_5 cluster and its ligand environment. **b** Hydrogen bond around Y_Z (D1-Tyr 161). **c** Hydrogen bond around Y_D (D2-Tyr 160). **d** The Q_A-bicarbonate (BCT)-Q_B site. **e** The P680 site.

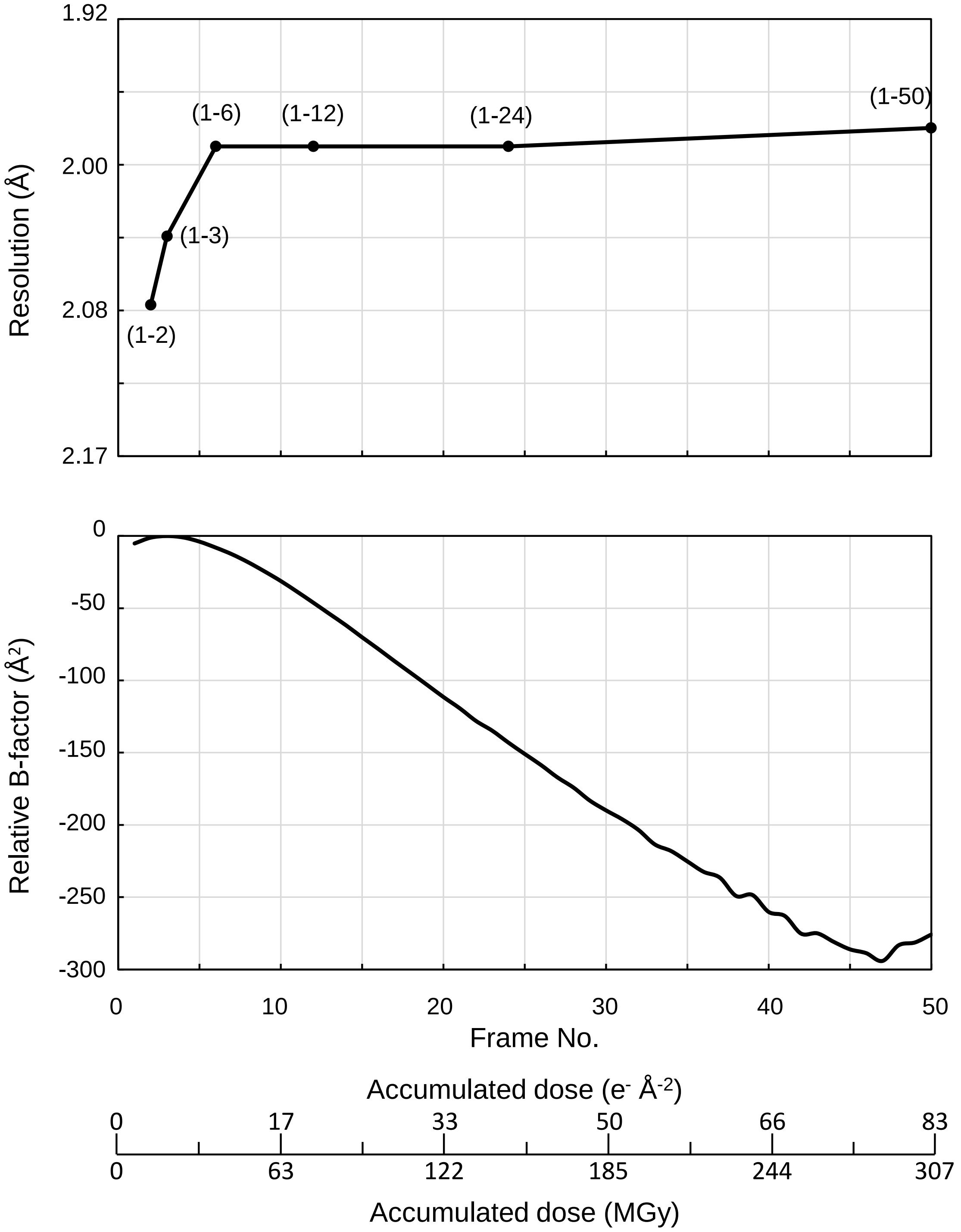

Supplementary Fig. 8 Plot of resolutions achieved against the electron doses (top) and per-frame B-factors (bottom). top, resolutions achieved by reconstructions using reduced frames of the total movies for the ARM-60k dataset. The parentheses in the graph indicate the number of frames. **bottom,** per-frame B-factors as calculated with the Bayesian polishing function of RELION.

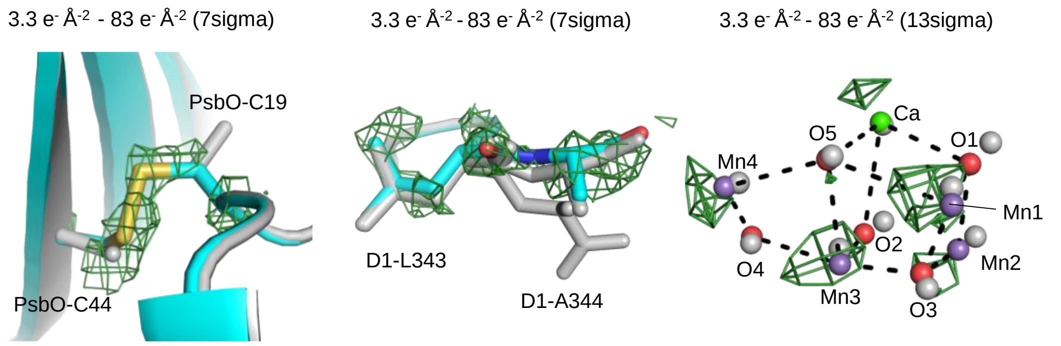

Supplementary Fig. 9 Difference maps at the damaged parts. The difference maps (low-dose map minus high-dose map) for each damaged area is displayed as a green mesh and the corresponding models for low-dose (colored) and high-dose (gray) are shown as sticks (see Methods).

Supplementary Table 1 Parameters for the cryo-EM data collection.

**Data set**	**Titan-75K**	**Titan-96K**	**ARM-60K**
Microscope	Titan Krios	Titan Krios	CRYO ARM 300
Detector	Falcon3EC in EC mode	Falcon3EC in EC mode	Gatan K2 summit in Counting mode
Total electron dose ($e^{-}Å^{-2}$)	40	40	83
Exposure time (s)	45.11	24.64	10.00
Dose rate ($e^{-}Å^{-2}\ s^{-1}$)	0.89	1.62	8.30
Number of frames per image	78	39	50
Dose per frame ($e^{-}Å^{-2}$)	0.51	1.03	1.66
Exposure time per frame (s)	0.58	0.63	0.20

Supplementary Table 2 Ligands of Chls and their distances in the high-dose, high resolution cryo-EM structure of PSII and their standard deviations.

	Chl	Ligand	Distance (Å)	Variance (Å)*
D1	405	H198	2.31	0.00774
	406	H_2O	2.18	0.00176
	407	H_2O	2.17	0.0027
	410	H118	2.17	0.0027
CP47	602	H_2O	2.18	0.00176
	603	H201	2.21	0.00014
	604	H202	2.16	0.00384
	605	H455	2.25	0.00078
	606	H100	2.23	0.00006
	607	H157	2.24	0.00032
	608	H_2O	2.14	0.00672
	609	H466	2.26	0.00144
	610	H216	2.29	0.00462
	611	H_2O	2.22	0
	612	H469	2.19	0.00102
	613	H23	2.19	0.00102
	614	H26	2.33	0.01166
	615	H9	2.28	0.00336
	616	H149	2.26	0.00144
	617	H114	2.25	0.00078
CP43	501	H237	2.23	0.00006
	502	H430	2.19	0.00102
	503	H118	2.25	0.00078
	504	H_2O	2.22	0
	505	H441	2.22	0
	506	H251	2.2	0.00048
	507	H_2O	2.13	0.00846
	508	H444	2.24	0.00032
	509	H53	2.15	0.00518
	510	H56	2.22	0
	511	N39	2.19	0.00102
	512	H164	2.2	0.00048
	513	H132	2.29	0.00462
D2	401	H197	2.29	0.00462
	403	H117	2.24	0.00032
Average			2.22	0.002324
S.D.				0.048

*Variance is the square of the difference between that particular distance and the average distance, and S.D. is the root of the average variance.
