# Supplementary material for: High-resolution cryo-EM structure of photosystem II reveals damage from high-dose electron beams
Source: Commun Biol. 2021 Mar 22;4:382. doi: 10.1038/s42003-021-01919-3 (PMC7985191; doi:10.1038/s42003-021-01919-3)
Supplement: Supplementary file 3 — Description of Additional Supplementary Files [file 42003_2021_1919_MOESM3_ESM.pdf]

Description of Additional Supplementary Files

File Name: Supplementary Data 1

Description: Contains source data for Fig. 1 used in the main text.
